# Observational Study on Variation of Longitudinal Platelet Counts in Calves over the First 14 Days of Life and Reference Intervals from Cross-Sectional Platelet and Leukocyte Counts in Dairy Calves up to Two Months of Age

**DOI:** 10.3390/ani11020347

**Published:** 2021-01-29

**Authors:** Emma Strous, Arne Vanhoudt, Anja Smolenaars, Gerdien van Schaik, Matthijs Schouten, Henrik de Pater, Bjorn Roelofs, Mirjam Nielen

**Affiliations:** 1 Department of Population Health Sciences, Faculty of Veterinary Medicine, Utrecht University, Yalelaan 7, 3584 CL Utrecht, The Netherlands; a.vanhoudt@uu.nl (A.V.); G.vanSchaik@uu.nl (G.v.S.); matthijsch@hotmail.com (M.S.); t.h.depater1989@gmail.com (H.d.P.); bjorn@dierenkliniekdenham.nl (B.R.); M.Nielen@uu.nl (M.N.); 2 Royal GD (Animal Health Service), Deventer, Arnsbergstraat 7, 7418 EZ Deventer, The Netherlands; anja.de.bont@crv4all.com

**Keywords:** platelets, leukocytes, reference interval, dairy calves, postnatal increase

## Abstract

**Simple Summary:**

To define a healthy animal in an experimental setting or to differentiate and backup a diagnosis in cattle practice, reference intervals (RIs) in haematology diagnostics are necessary. The RIs in calves for blood cell counts, such as platelets and white blood cells, differ from RIs in adult cattle and are not widely studied. Blood results from dairy calves in the Netherlands were used to study the variation in platelet counts in young calves and to calculate an RI for platelet and white blood cell counts. In new-born calves up to six days of age, platelet counts were lower than in calves older than five days. From six days of age until 60 days of age we propose an RI platelet count of 287–1372 × 10^9^/L and for the first 60 days of life an RI for leukocyte count of 4.0–18.9 × 10^9^/L.

**Abstract:**

Platelet and leukocyte count reference intervals (RIs) for cattle differ by age and while adult RIs are known, RIs for calves are studied less. The aims of this observational study are to evaluate variation of platelet counts of Holstein Friesian calves over the first 14 days of life and to propose RIs for platelet and leukocyte counts of Holstein Friesian calves aged 0–60 days. In a longitudinal study, 19 calves were blood sampled 17 times, in the first 14 days of their lives. Blood was collected in a citrate blood tube and platelet counts were determined. We assessed the course of platelet counts. In a field study, 457 healthy calves were blood sampled once. Blood was collected in an EDTA blood tube and platelet and leukocyte counts were determined. The RIs were calculated by the 2.5 and 97.5 percentiles. Platelet counts started to increase 24 h after birth (mean platelet count 381 × 10^9^/L ± 138 × 10^9^/L) and stabilized after five days (mean platelet count 642 × 10^9^/L ± 265 × 10^9^/L). In calves up to six days of age, platelet counts were lower than in calves older than five days. In conclusion, the RIs of platelet and leukocyte counts in calves were wider in range than the RIs for adult cattle, therefore, calf specific RIs for platelet and leukocyte counts should be used. From 6 until 60 days of age, we propose an RI for platelet counts of 287–1372 × 10^9^/L and for the first 60 days of life an RI for leukocyte counts of 4.0–18.9 × 10^9^/L.

## 1. Introduction

To define a healthy animal in an experimental setting or to differentiate and backup a diagnosis in cattle practice or in specialized bovine clinics, reference intervals (RIs) in haematology diagnostics are necessary. The RIs for blood cell counts, such as platelets and leukocytes, are widely available for adult cattle. However, RIs are less abundant for calves and furthermore, are known to differ greatly for calves [1,2]. Reference intervals for platelet and leukocyte counts in adult cattle range between 155–1022 × 10^9^/L and 4.9–14.3 × 10^9^/L, respectively [3,4]. While haematologic RIs in calves have been studied, they are often based on a small sample size [5,6], focus on a very specific age range [7] or use older analytical techniques [8]. Blood samples can be collected in different blood tubes for platelet and leukocyte counts. The ethylenediaminetetraacetic acid (EDTA) blood tube is mostly used in practice, while citrate tubes are often used in experimental studies.

When bovine neonatal pancytopenia (BNP) started to appear in Western Europe in 2007, RIs for platelet and leukocyte counts in calves were unavailable, which made it difficult to confirm the diagnosis of thrombocytopenia. Additionally, knowledge was lacking on whether, and how, platelet counts in calves change after birth. BNP was likely caused by maternal antibodies in colostrum from dams vaccinated with PregSure^®^BVD (Pregsure, Pfizer Animal Health) [9]. Clinical signs of BNP in calves occurred within 10–20 days of age and consisted of petechiae, cutaneous bleeding, melena and death within five days in 90% of the calves [9,10]. Haematologic findings in BNP calves included thrombocytopenia and leukopenia, and bone marrow depletion upon histopathology.

We used two datasets from the BNP period to study platelet and leucocyte counts in calves. The aims of this observational study were (1) to evaluate platelet count variation from a dataset of healthy calves aged 0–14 days in a longitudinal study under controlled circumstances using citrate blood tubes in a ruminant clinic, and (2) to propose RIs for platelet and leukocyte counts using EDTA blood tubes from a dataset of calves aged 0–60 days in a cross-sectional field study in the Netherlands. The expectations were that calves would have different platelet and leukocyte count RIs compared to adults and that there might be variation in platelet counts in the first two weeks of age.

## 2. Materials and Methods

### 2.1. Ethic Statements

All procedures in the longitudinal study were in agreement with the Dutch Act on Animal Experimentation and approved by the local ethics committee of Utrecht University (DEC number 2011.iii.01.021).

All procedures in the field study were performed in conformance with European law (Directive 86/609/EEC) and were considered standard diagnostic procedure.

### 2.2. Longitudinal Study

#### 2.2.1. Animal Recruitment

Nineteen pregnant Holstein Friesian heifers from 11 different farms were bought into the Utrecht University ruminant clinic for an unrelated project. The heifers were selected on absence of previous disease and absence of PregSure^®^BVD (Pfizer GmbH, Berlin, Germany) vaccination. Calves were born via normal parturition without complications (*n* = 17), after a corrected uterine torsion (*n* = 1) or via a caesarean section (*n* = 1). Calves were sold to the veal industry after reaching 14 days of age.

#### 2.2.2. Inclusion and Exclusion Criteria

All 19 Holstein Friesian calves (9 female, 10 male) born were included in the study. Calves observed with disease were highlighted in the dataset on the day(s) of disease as well as two days earlier and afterwards. Diseased calves included calves with pyrexia (rectal temperature > 39.5 °C), respiratory (nasal discharge, increased breathing frequency, increased breathing effort and/or coughing) or gastro-intestinal (diarrhoea) disease, or a combination of these and standard clinic therapies were provided.

#### 2.2.3. Blood Sample Collection and Handling

Venous jugular blood samples were collected by veterinary students using a Vacutainer, 21 G needles and plastic evacuated tubes containing coagulation sodium citrate 3.2% (9 NC Coagulation sodium citrate 3.2%, Greiner bio-one), mixing ratio one-part citrate solution, nine parts blood. Blood tubes were filled to exclude the variance in dilution effect of the citrate. Blood samples were obtained immediately after birth (T = 0 h) and at the following 1, 2, 3, 6, 12, 24 h and 2, 3, 4, 5, 6, 7, 8, 10, 12, 14 days of age. The time of sampling, except during the first 24 h of life, was between 9 and 10 a.m. Blood samples were transported to the University Veterinary Diagnostic Laboratory (UVDL) by a pneumatic tube transport system and immediately refrigerated at 5 °C until processing, which would be within four hours during working hours and could be up to 72 h if the sample was collected at the beginning of the weekend. Platelet counts were measured with the haematology analyser ADVIA 120 (Siemens Healthcare Diagnostics B.V., Breda, The Netherlands) following the operator manual. Measurements were looked at and all platelet concentrations smaller than 100 × 10^9^/L were checked manually in a blood smear to exclude platelet aggregation. Besides that, the haematology analyser ADVIA 120 has an internal alarm for platelet aggregation. With a reported coefficient of variation (CV) of 4.7% for cattle [3], the total observed error (TEobs = 2 CV + unknown bias) of the ADVIA 120 is likely to have remained below the total allowable error of 20% [11].

#### 2.2.4. Housing and Feeding

Calves were housed in individual indoor calf boxes bedded with straw. Every calf was provided with two litres of its own dam’s colostrum within two hours from birth as well as at 6 and 12 h of age. When this was unavailable or the quality, as determined by colostrometry (KRUUSE UK Ltd., Colostrum Densimeter, Langeskov, Denmark), was low (i.e., specific gravity < 1035), frozen colostrum of confirmed good quality (i.e., specific gravity > 1045), previously obtained from dams not having been exposed to PregSure^®^BVD, was used. In case the calf would not drink voluntarily, it was fed with an oesophageal tube. After the colostrum intake, milk replacer (Fokkamel Plus^®^. De Heus Voeders, Ede, The Netherlands; 21% protein) was fed (two litres, three times daily), prepared according to manufacturer’s instructions (150 g of powder was supplemented to water to make one litre milk replacer). Calves had ad libitum access to water from birth. During the 14-day study, calves did not receive hay or concentrates.

### 2.3. Field Study

#### 2.3.1. Setting

The field study was carried out using blood samples and farm and animal data collected in 2011 during the study by Jones et al. [12], which investigated risk factors associated with BNP. The data were collected in the Netherlands, France, Germany and Belgium. In our RI study, only the data from the Netherlands were used.

#### 2.3.2. Animal Recruitment

Details of animal recruitment in the field study are described by Jones et al. [12]. In brief, on farms with a suspected BNP case, up to four calves that were determined clinically healthy by veterinary examination and of similar age as the animal presumably affected by BNP, were recruited as control animals and blood sampled using EDTA blood tubes. Full clinical examination and blood sampling of the calves was performed by either local veterinary practitioners or a veterinarian from the research team. When control calves reached 28 days of age, the farmers were contacted again by the research team of Jones et al. [12] to confirm that these calves had not developed clinical signs of BNP, other diseases or had been medicated. In addition, the identification and registration system was consulted to determine whether the control calves were still alive.

Calves were used for normal commercial purposes after the end of the study period: female calves stayed on the farm, while male calves were generally sold to the veal industry at the age of 28 days.

#### 2.3.3. Inclusion and Exclusion Criteria

**Figure 1:**
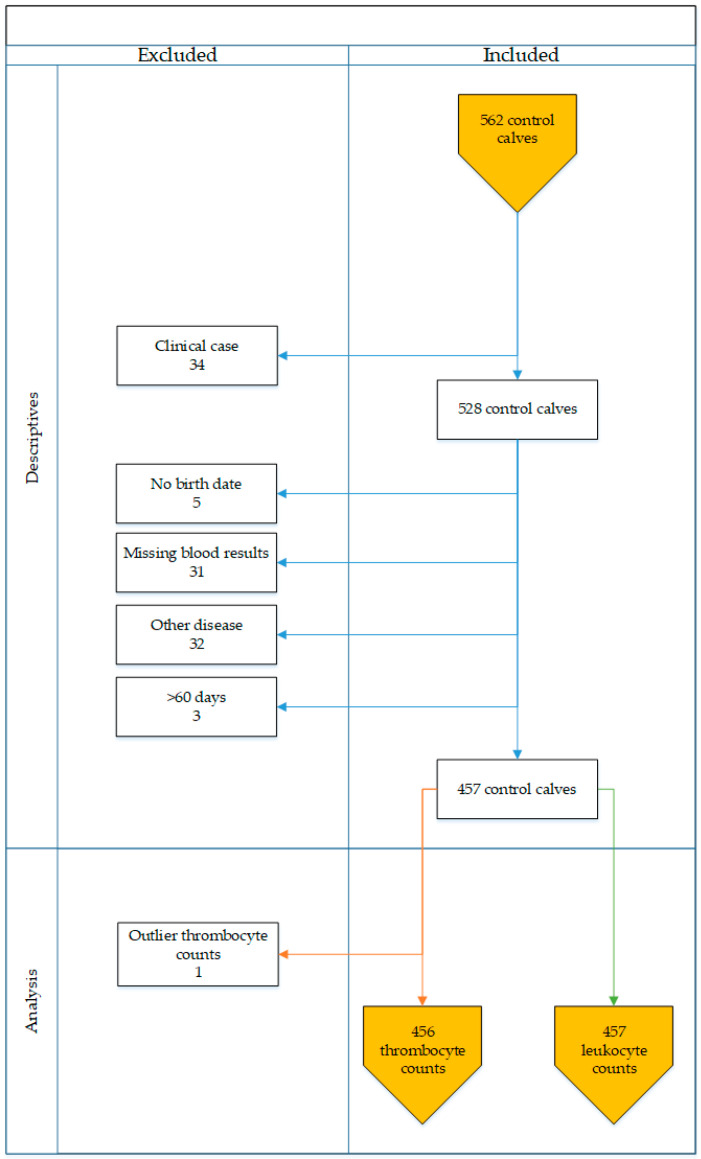
Schematic overview of calves included and excluded from the field study.

Selection of animals from the group of control calves of the study by Jones et al. [12] for the current RI field study dataset was based on three inclusion criteria: (1) the calf did not develop clinical signs of BNP between the moment of sampling and reaching 28 days of age, (2) platelet and leukocyte count results were available and (3) the calf was aged between 0 and 60 days at the moment of sampling, leading to a larger amount of blood samples compared to the original study by Jones et al. [12] (Figure 1). In total, 457 predominantly Holstein Friesian (87%) calves (340 female, 105 male and 12 calves of unrecorded sex) aged 0–60 days were enrolled in this study (Table 1). Thirteen percent of the calves were of mixed breed: Holstein with Meuse-Rhine-Yssel (MRY), Jersey or Fleckvieh. Calves enrolled originated from 135 dairy farms: one, two, three or four calves were sampled on four, 15, 41 and 75 farms, respectively.

#### 2.3.4. Blood Sample Collection and Handling

Venous jugular blood samples were collected by local veterinarians, who were financially compensated for their work, or a veterinarian from the research team of Jones et al. [12]. Samples were collected into vacutainer tubes of 10 mL, which contained 18.0 mg K2 potassium salt of ethylenediaminetetraacetic acid and a minimum of a half-filled EDTA tube was required for analysis. The chilled blood samples were transported by courier from veterinary practices to Royal GD in Deventer, the Netherlands for analysis of the blood cells. Analysis of the samples took place on the day of arrival at the laboratory. The platelet and leukocyte counts were determined using an automatic haematology analyser (Cell-Dyn 3700; Abbot Laboratories, Abbot Park, IL, USA), following the operator manual. Platelet aggregation was not assessed and information regarding the CV and TEobs were not available.

### 2.4. Statistical Analyses

Farm data, animal data, platelet and leukocyte counts were analyzed in SPSS (version 25, IBM SPSS Data Collection).

#### 2.4.1. Longitudinal Study

Platelet counts of diseased animals, including the platelet counts of the two days before and after clinical disease, were excluded from the analysis. Platelet counts were plotted in boxplots and a scatter plot to determine outliers (<2.5 th percentile or >97.5 th percentile) and to visually assess concentrations of platelets over time. Data were analyzed for normality using the Shapiro-Wilk test. A mixed multilevel model analysis for platelet counts was performed on the data for three variables: sex, time of sampling and delay in sample analysis. Blood samples taken in the weekend or after 3 pm on a Friday were delayed in sample analysis at the UVDL. Platelet counts of 70 samples (29%) were delayed. Calf ID was used as random effect. A *p* < 0.05 was considered significant.

#### 2.4.2. Field Study

Platelets counts and leukocytes counts were analyzed for normality using the Shapiro-Wilk test. A histogram and the Q-Q Plot of platelet counts had one visual outlier, confirmed with Dixon’s criterion [13,14]. The outlier was excluded from further analysis. A histogram and Q-Q plot of leukocyte counts had two visual outliers after log transformation (ln). However, the outliers could not be confirmed with Dixon’s criterion. Before calculations of the RIs, two comparisons were tested: one to determine age effect and one to determine sex effect. To determine a possible age effect, platelet and leukocyte counts were arranged by age in weeks. Number of counts varied per week; most were week 2 (*n* = 142). The number of samples per week are presented in Table 1. The numbers of samples obtained in week 5, 6, 7, 8 and 9 were small, which is why those weeks were pooled to one group (*n* = 72). The counts in those new groups were not all normally distributed according to the Shapiro-Wilk test. The comparison between week 1, 2, 3, 4 and the combined weeks 5–9 was conducted by a Kruskal-Wallis analysis of variance. To determine a possible sex effect, platelet and leukocyte counts of male calves (*n* = 104) were compared to female calves (*n* = 271) using a Wilcoxon rank sum test. Animals up to four weeks of age were used because after that age, the number of male animals on farms decreased drastically as male calves were sold to the veal industry. The RIs of platelet and leukocyte counts for all calves, aged 0–60 days, were determined by the 2.5 and 97.5 percentiles including the 90% confidence interval (CI) of these limits [13,15,16].

## 3. Results

### 3.1. Longitudinal Study

Nineteen calves were eligible for blood sampling at 17 time points. A maximum of 16 calves and minimum of 12 calves were sampled per time point. Having fewer than 19 samples per time point resulted from missing blood results, e.g., due to blood clotting (*n* = 6) or being excluded due to calves marked as diseased (*n* = 74). In total, 243 platelet counts were included.

The mean platelet count was 435 × 10^9^ (SD = 175) at birth and 743 × 10^9^ (SD = 291) on day 6 (144 h) (Table 2). All platelet counts per time point, except for the platelet counts at 72 h, were normally distributed.

**Figure 2:**
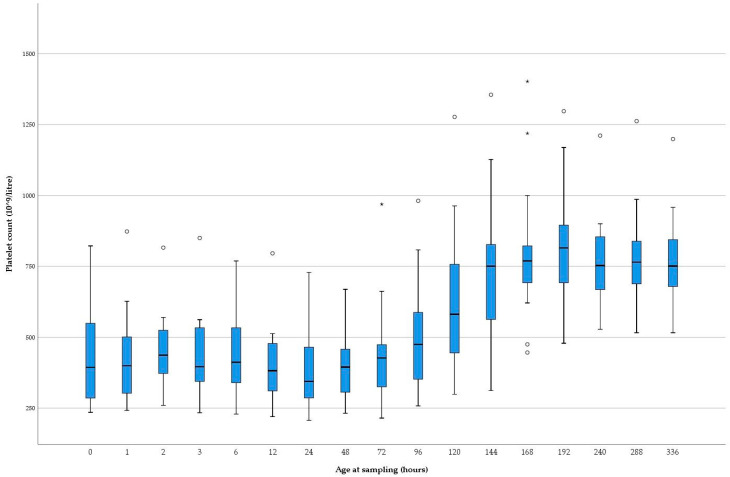
Boxplots of platelet counts (×10^9^/L) per sampling point in the longitudinal study. Blue box representing the central 50% of the platelet counts. The median is marked by the horizontal black line inside of the blue box. The whiskers define the 2.5th and 97.5th percentiles. ° = outlier outside the ranges 3rd quartile + 1.5× interquartile range or 1st quartile–1.5× interquartile range. * = outlier outside the ranges 3rd quartile + 3× interquartile range.

**Figure 3:**
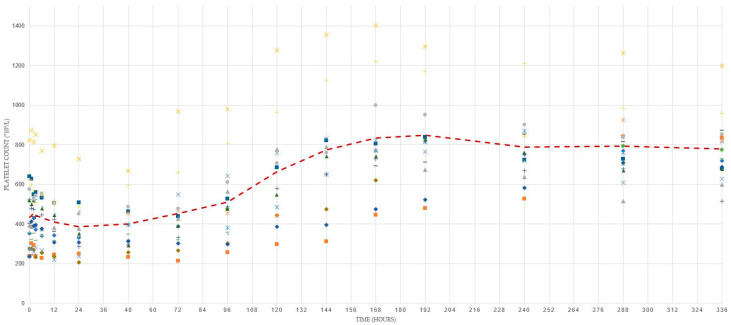
Scatter plot of platelet counts (×10^9^/litre) per calf over time in the longitudinal study and a dotted red line representing the mean platelet counts. Each symbol represents consecutive values from an individual calf.

Boxplots of the platelet counts of all calves visualized a few outliers (Figure 2). Four calves were identified as outliers at least once. For example, the platelet counts of one calf were an outlier at several sampling times. However, those platelet counts followed the same trend line when compared to the platelet counts of the other animals (Figure 2). Platelet counts followed a similar pattern over time in all calves with an increase in platelet count beginning between 48 and 168 h (two and seven days) of age (Figure 3).

Platelet counts sampled in calves younger than six days of age (birth-5 days old) were lower than those from calves older than five days (>5 days old) (*p* < 0.001). Platelet counts did not differ between male and female calves (*p* = 0.38), nor between samples with delayed and timely analysis (*p* = 0.50).

### 3.2. Field Study

#### 3.2.1. Platelet Counts

Descriptive statistics of platelet counts are presented in Table 3. The mean platelet count was 787 × 10^9^/L (SD = 269), with a minimum of 132 × 10^9^/L, a maximum of 1663 × 10^9^/L and a range of 1531 × 10^9^/L. The platelet counts were not normally distributed (*p* = 0.001).

Platelet counts of week 1, 2 and the combined weeks 5–9 did not differ from each other (all *p* > 0.05). Platelet counts of week 3 differed from those of week 4 (*p* = 0.03).

Platelet counts were not different between male (*n* = 104) and female (*n* = 271) calves within the first four weeks of life (*p* = 0.36).

As our longitudinal study data indicate that platelet counts during the first five days of life are lower when compared with those from calves aged 14 days, inclusion of the platelet counts of the first five days of age might cause underestimation of the lower RI limit for older calves. When we excluded the platelet counts of the first five days in the field study (*n* = 21 counts), the RI for platelet counts was 287–1372 × 10^9^/L (Table 3) with a 90% CI of 182–356 × 10^9^/L for the lower limit and 1322–1568 × 10^9^/L for the upper limit.

#### 3.2.2. Leukocyte Counts

Descriptive statistics of leukocyte counts are presented in Table 3. The mean leukocyte count was 9.3 × 10^9^/L (SD = 1.5), with a minimum of 2.7 × 10^9^/L, maximum of 37.0 × 10^9^/L and a range of 34.3 × 10^9^/L. The leukocyte counts were not normally distributed (*p* < 0.001).

Leukocyte counts of week 1, 2, 3, 4 and the combined weeks 5–9 did not differ from each other (*p* = 0.66). Leukocyte counts did not differ between male (*n* = 104) and female (*n* = 271) calves within the first four weeks of life (*p* = 0.12).

The RI of leukocyte counts for calves between 0–60 days was 4.0–18.9 × 10^9^/L (Table 3) calculating the 2.5 and 97.5 percentiles with a 90% CI of 3.6–4.6 × 10^9^/L for the lower limit and 17.6–20.9 × 10^9^/L for the upper limit.

## 4. Discussion

For platelet and leukocyte counts in neonatal calves, RIs have been published before [5,6,7,8,17,18]. We analysed the changes in platelet count in the first two weeks of life under semi-controlled circumstances and used field data to determine platelet and leukocyte count RIs. Using field data to determine RIs will inevitably include more variation and is not considered a gold standard. However, in the absence of precise RIs determined by an experimental study, our proposed RIs provide a good estimate of the actual platelet and leukocyte counts in dairy calves and might be a better reflection of the dairy calf population, which is predominantly Holstein-Friesian, Red Holstein Friesian or their crossbreds. Moreover, our study includes more calves than previous studies.

While monitoring platelet counts during the first two weeks of life, lower counts were observed in counts from calves in the first five days of life, in comparison with those from calves older than five days. The rise in platelet counts during the first week of life was described earlier by others [5,6,18,19], although in those studies calves were sampled for the second time after a week and not sampled every day like in our study.

A lower concentration of platelets in general can be caused by reduced production, excessive use, breakdown, sequestration or loss. In neonatal calves, sequestration of platelets might happen through accumulation around the navel in the umbilical cord, as they are needed there at birth when the umbilical cord breaks. Furthermore, counting the actual concentration of platelets might be impossible due to their lack of maturation [20].

Unfortunately, the counts of leukocytes and other blood cells were not available from the longitudinal study. Some have described that leukocyte counts were highest at birth and then decreased, but there is no consensus on when the leukocyte count stabilises, ranging from three to 84 days of age [5,8,21]. Others have described that leukocyte counts oscillated slightly throughout the first 24 weeks of life [18].

From our field study, the platelet and leukocyte count RIs and the higher RI upper limits in calves than in cows, are comparable to results from Panousis et al. [7] (platelet count RI: 213.8–1251.0 × 10^9^/L, leukocyte count RI: 3.84–19.55 × 10^9^/L) who also used the 2.5 and 97.5 percentiles. Panousis et al. [7] speculated that calves might have higher thrombopoietin concentrations compared to adult cows as is the case in human new-borns [22,23]. The higher RI upper limits are also in agreement with Brun-Hansen [6] who found that the platelet count RI upper limit for calves until the age of 19–21 weeks is above that for adult Norwegian Red cattle. However, Brun-Hansen et al. [6] report calves to have much narrower RIs for platelet counts (518–987 × 10^9^/L) and leukocyte counts (9.4–12.0 × 10^9^/L). The lower limits of the RIs in particular were remarkably high compared to our findings. This might be explained by their RI calculation method (unknown), smaller sample size (15 calves) or breed (Norwegian Reds). The difference in sample size seems the more plausible of these, given that adult Norwegian Reds seem to have platelet count (200–590 × 10^9^/L) and leukocyte count (4.7–11.4 × 10^9^/L) RIs similar to those used for adult cattle by the laboratory of Royal GD (platelet count RI: 193–637 × 10^9^/L; leukocyte count RI: 4.9–12.0 × 10^9^/L) [4].

Our results differ from Mohri et al. [5], who reported that the platelet count RI of calves between 1–84 days of age was consistent with the RI in adult cattle (100–800 × 10^9^/L [24,25]). This might be explained by the different calculation methods, as their results are presented in a graph using mean (SE) values of platelet counts to compare to the RI of adult cattle. Based on our and other studies, having a dairy calf RI for platelet counts and leukocyte counts is justified.

The platelet count RI in our field study had higher lower and upper limits than RIs for adult cattle used in both laboratories [3,4]. The RI for leukocyte counts in our field study was wider compared with RIs for adult cattle used in both laboratories [3,4]. The platelet and leukocyte count RIs, determined by George et al. [4], were calculated by mean ± 2 SD, which is different from our method. RI calculation methods differ dependent on sample size and the shape of the distribution of the data [13,16]. Calculating the RIs with the SD method for our data gave only slightly different RIs compared to our calculation method: platelet count RI of 276–1319 × 10^9^/L and leukocyte count RI of 4.5–19.5 × 10^9^/L. The wider range of RIs might be caused by a real difference in platelet and leukocyte counts between calves and adult cattle or may be due to sample size: George et al. [4] used 58 cattle compared to the 456 and 455 calves in our RI analysis.

Some might suggest pre-pancytopenia or subclinical disease could have been the cause of the wider platelet and leukocyte counts RI of apparently healthy animals. This could be suspected especially because in the field study, control calves were chosen if there was a calf with clinical signs of BNP on the same farm. However, to minimize the chance of including calves with pre-pancytopenia or subclinical disease into the study, the data of the calves with the highest values of the leukocyte counts and the outlier for platelet counts were studied with more detail. No pattern was identified in sample dates, season or farm.

The calf with the outlier platelet count had a high platelet count rather than a low one, which makes pre-pancytopenia unlikely. In addition, considering the follow-up after 28 days of age, calves with pre-pancytopenia would have eventually become BNP affected, died due to the high mortality of the disease and been excluded from the RI field study. Moreover, as the field study took place during a BNP outbreak with high impact, we expect that the farmers were likely better and more often inspect their youngstock for diseased calves.

In humans, a lower initial platelet count in neonates followed by an increase [26] and age differences in platelet counts [27] are seen as well. During human embryogenesis and postnatal development thrombopoiesis occurs at distinct sites. Foetal liver megakaryocytes generate and release platelets that differ functionally from platelets from bone marrow megakaryocytes [28]. The switch in production site might be a cause for the initial lower platelet count. Several differences in platelet production between human neonates and adults are known: (1) thrombopoietin concentrations are higher in neonates than in healthy adults [28,29,30], (2) neonatal megakaryocyte progenitors proliferate faster than adult ones [29], but are (3) significantly smaller [31] and (4) neonatal megakaryocytes produce fewer platelets per cell [32].

The neonatal platelet production and turnover were investigated in a mouse model by Liu et al. [33]. They found that new-born and adult mice had similar platelet production rates, but neonatal platelets survived one day longer in circulation. The prolonged lifespan fully accounted for the rise in platelet counts observed during the second week of murine postnatal life [33]. Hypotheses for the age-dependent reduction in platelet counts include a decline in hematopoietic stem cell reserves during aging or a survival advantage for adult subjects with low platelet counts [27]. It is unknown if these hypotheses also hold true for cattle.

The blood tubes used in our studies differ. Citrate tubes were used in the longitudinal study and EDTA tubes were used in the field study. EDTA tubes are most commonly used in veterinary practice to determine platelet counts, especially when a complete blood count is performed. However, citrate tubes can be used too and are often used for research purposes.

For platelet counts, the EDTA tube is advised by the International Council for Standardization for Haematology [34]. A risk of using EDTA in a tube, is that it might cause agglutination of platelets, resulting in pseudothrombocytopenia [35,36]. The platelet counts might have been underestimated in some samples of the field study as platelet clumping was not assessed and might partly explain the somewhat wide platelet count RI. According to George [37], many large (human) studies have shown that falsely low platelet counts, in most cases due to platelet agglutination caused by EDTA, occurs in about one person in 1000, irrespective of the presence or absence of any disease. Although EDTA-dependent pseudothrombocytopenia could have happened in the samples from the field study, we do not think that this had major effects on the proposed RI.

A disadvantage of citrate tubes could be that the platelet concentration decreases during storage [38]. We compared the platelet counts in five calves using both a citrate and an EDTA tube of each calf. A decrease in platelet count after 72 h of storage could not be detected (results are available upon request). The maximum time of storage in the laboratory was 72 h, so the use of citrate blood tubes was deemed reliable.

## 5. Conclusions

In general, the RIs of platelet and leukocyte counts found in calves were wider in range than RIs for adult cattle, therefore, calf specific RIs for platelet and leukocyte counts should be used. Although the studies differ completely, together they describe the course of platelet counts during the first two weeks of age and provide an RI for platelet and leukocyte counts in young calves.

Calves under six days of age showed low and variable platelet counts, hereafter platelet counts stabilized. Hence, we cannot propose a platelet count RI for the first five days of age. From six days old until 60 days of age we propose an RI for platelet counts of 287–1372 × 10^9^/L and for the first 60 days of life an RI for leukocyte counts of 4.0–18.9 × 10^9^/L.

## Figures and Tables

**Table 1:** Distribution of platelet and leukocyte counts of calves at age of sampling and sex, in the field study.

Week Number (Age in Days)	Total Amount	Male	Female	Sex Unrecorded
1 (0–7)	39	15	19	5
2 (8–14)	142	60	81	1
3 (15–21)	105	17	84	4
4 (22–28)	99	12	87	0
5 (29–35)	34	}1	}69	}2
6 (36–42)	21			
7 (43–49)	12			
8 + 9 (50–60)	5			

**Table 2:** Descriptive results of 243 platelet counts (×10^9^/L) from calves in the longitudinal study. N = amount of calves, M = amount of male calves, F = amount of female calves, SD = standard deviation, Min. = minimum, Max. = maximum, CI = confidence interval.

Age at Blood Sampling (Hours)	N	M	F	Mean	Mean Male	Mean Female	Median	SD	Min.	Max.	Range	95% CI
0	15	7	8	435	471	403	394	175	235	822	587	338–532
1	14	7	7	439	485	394	400	172	242	873	631	340–539
2	15	7	8	453	493	419	437	143	260	816	556	374–532
3	15	7	8	440	473	411	396	158	234	850	616	352–528
6	14	6	8	429	470	398	412	147	229	769	540	344–531
12	15	7	8	403	444	368	382	147	220	796	576	322–485
24	14	6	8	381	441	335	345	138	207	729	522	301–461
48	15	7	8	395	439	356	395	123	232	669	437	326–463
72	15	7	8	443	494	398	427	184	215	969	754	341–545
96	16	8	8	498	519	477	475	194	258	981	723	395–602
120	13	6	7	642	685	605	581	265	299	1277	978	482–803
144	12	5	7	743	819	689	751	291	312	1355	1043	558–928
168	13	6	7	807	864	759	769	268	446	1402	956	645–969
192	12	5	7	822	899	767	815	235	479	1297	818	673–972
240	13	5	8	773	833	736	753	173	528	1211	683	669–878
288	16	7	9	787	907	694	765	171	516	1262	746	696–879
336	16	7	9	773	877	691	751	161	516	1199	683	687–859

**Table 3:** Reference intervals (RIs) of platelet and leukocyte counts of calves 0–60 days, calves 6–60 days and descriptive results derived from the field study. All values shown in ×10^9^/L.

RIs and Descriptives	Platelet Count (×10^9^/L)	Leukocyte Count (×10^9^/L)
	*n* = 435	*n* = 457
RI calves 0–60 days		4.0–18.9
RI calves 6–60 days	287–1372	
Descriptive results	*n* = 456	*n* = 457
Mean	787	9.3
Median	773	9.3
Standard Deviation	269	1.5
Minimum	132	2.7
Maximum	1663	37.0
Range	1531	34.3

## Data Availability

Data of the longitudinal study are available from the authors upon reasonable request. There are legal restrictions on sharing the data set of the field study. The data were collected within a field study at commercial farms. The farmers signed an informed consent that included an explicit statement about future use of the data (translated from Dutch) ’The data will be used solely for the Dutch part of the project on BNP and will never be made available to third parties’. The laboratory tests were carried out at Royal GD, leading to ownership of these laboratory results, that however cannot legally be provided to third parties because of the informed consent statement. In the current project, the original researchers were involved and agreed that the data could be used for this research project. We see a similar situation for future requests by third parties: the data can be used if and when the research questions would fit within the original BNP research goal AND the data are analysed anonymously, within the Netherlands. The official contact will be Royal GD at: info@gddiergezondheid.nl.

## References

[1] Witt K., Weber C.N., Meyer J., Buchheit-Renko S., Müller K.E. (2011). Haematological analysis of calves with bovine neonatal pancytopenia. Vet. Rec..

[2] Meyer D.J., Harvey J.W. (2004). Interpretation and Diagnosis. Veterinary Laboratory Medicine.

[3] Moritz A. (2002). Der Einsatz Lasergestützter Multiparameter-Hämatologiesysteme in der Veterinärmedizin.

[4] George J.W., Snipes J., Lane V.M. (2010). Comparison of bovine hematology reference intervals from 1957 to 2006. Vet. Clin. Pathol..

[5] Mohri M., Sharifi K., Eidi S. (2007). Hematology and serum biochemistry of Holstein dairy calves: Age related changes and comparison with blood composition in adults. Res. Vet. Sci..

[6] Brun-Hansen H.C., Kampen A.H., Lund A. (2006). Hematologic values in calves during the first 6 months of life. Vet. Clin. Pathol..

[7] Panousis N., Siachos N., Kitkas G., Kalaitzakis E., Kritsepi-Konstantinou M., Valergakis G.E. (2018). Hematology reference intervals for neonatal Holstein calves. Res. Vet. Sci..

[8] Tennant B., Harrold D., Reina-Guerra M., Kendrick J.W., Laben R.C. (1974). Hematology of the neonatal calf: Erythrocyte and leukocyte values of normal calves. Cornell Vet..

[9] Bastian M., Holsteg M., Hanke-Robinson H., Duchow K., Cussler K. (2011). Bovine Neonatal Pancytopenia: Is this alloimmune syndrome caused by vaccine-induced alloreactive antibodies?. Vaccine.

[10] Pardon B., Steukers L., Dierick J., Ducatelle R., Saey V., Maes S., Vercauteren G., De Clercq K., Callens J., De Bleecker K. (2010). Haemorrhagic diathesis in neonatal calves: An emerging syndrome in Europe. Transbound. Emerg. Dis..

[11] Friedrichs K.R., Harr K.E., Freeman K.P., Szladovits B., Walton R.M., Barnhart K.F., Blanco-Chavez J. (2012). ASVCP reference interval guidelines: Determination of de novo reference intervals in veterinary species and other related topics. Vet. Clin. Pathol..

[12] Jones B.A., Sauter-Louis C., Henning J., Stoll A., Nielen M., Van Schaik G., Smolenaars A., Schouten M., Den Uijl I., Fourichon C. (2013). Calf-level factors associated with bovine neonatal pancytopenia—A multi-country case-control study. PLoS ONE.

[13] Ceriotti F., Hinzmann R., Panteghini M. (2009). Reference intervals: The way forward. Ann. Clin. Biochem..

[14] Dixon W.J. (1953). Processing Data for Outliers. Biometrics.

[15] Petrie A., Watson P. (2013). Statistics for Veterinary and Animal Science.

[16] Horn P.S., Pesce A.J. (2003). Reference intervals: An update. Clin. Chim. Acta.

[17] Knowles T.G., Edwards J.E., Bazeley K.J., Brown S.N., Butterworth A., Warriss P.D. (2000). Changes in the blood biochemical and haematological profile of neonatal calves with age. Vet. Rec..

[18] Ježek J., Nemec M., Starič J., Klinkon M. (2011). Age Related Changes and Reference Intervals of Haematological Variables in Dairy Calves. Bull. Vet. Inst. Pulawy.

[19] Egli C.P., Blum J.W. (2010). Clinical, Haematological, Metabolic and Endocrine Traits During the First Three Months of Life of Suckling Simmentaler Calves Held in a Cow-Calf Operation. J. Vet. Med. Ser. A.

[20] Sola-Visner M. (2012). Platelets in the neonatal period: Developmental differences in platelet production, function, and hemostasis and the potential impact of therapies. Hematol. Am. Soc. Hematol. Educ. Progr..

[21] Zanker I.A., Hammon H.M., Blum J.W. (2001). Delayed feeding of first colostrum: Are there prolonged effects on haematological, metabolic and endocrine parameters and on growth performance in calves?. J. Anim. Physiol. Anim. Nutr..

[22] Walka M.M., Sonntag J., Dudenhausen J.W., Obladen M. (1999). Thrombopoietin concentration in umbilical cord blood of healthy term newborns is higher than in adult controls. Biol. Neonate.

[23] Wiedmeier S.E., Henry E., Sola-Visner M.C., Christensen R.D. (2009). Platelet reference ranges for neonates, defined using data from over 47,000 patients in a multihospital healthcare system. J. Perinatol..

[24] Radostits O.M., Blood D.C., Gay C.C. (2007). Veterinary Medicine: A Text Book of the Disease of Cattle, Sheep, Pigs, Goats and Horses.

[25] Latimer K.S., Mahaffey E.D., Prasse K.W. (2003). Duncan’s and Prasse’s Veterinary Labortory Medicine: Clinical Pathology.

[26] Henry E., Christensen R.D. (2015). Reference Intervals in Neonatal Hematology. Clin. Perinatol..

[27] Biino G., Santimone I., Minelli C., Sorice R., Frongia B., Traglia M., Ulivi S., Di Castelnuovo A., Gögele M., Nutile T. (2013). Age- And Sex-Related Variations in Platelet Count in Italy: A Proposal of Reference Ranges Based on 40987 Subjects’ Data. PLoS ONE.

[28] Andres O., Schulze H., Speer C.P. (2015). Platelets in neonates: Central mediators in haemostasis, antimicrobial defence and inflammation. Thromb. Haemost..

[29] Liu Z.-J., Italiano J., Ferrer-Marin F., Gutti R., Bailey M., Poterjoy B., Rimsza L., Sola-Visner M. (2011). Developmental differences in megakaryocytopoiesis are associated with up-regulated TPO signaling through mTOR and elevated GATA-1 levels in neonatal megakaryocytes. Blood.

[30] Ishiguro A., Nakahata T., Matsubara K., Hayashi Y., Kato T., Suzuki Y., Shimbo T. (1999). Age-Related changes in thrombopoietin in children: Reference interval for serum thrombopoietin levels. Br. J. Haematol..

[31] Ma D.C., Sun Y.H., Chang K.Z., Zuo W. (2009). Developmental change of megakaryocyte maturation and DNA ploidy in human fetus. Eur. J. Haematol..

[32] Mattia G., Vulcano F., Milazzo L., Barca A., Macioce G., Giampaolo A., Hassan H.J. (2002). Different ploidy levels of megakaryocytes generated from peripheral or cord blood CD34^+^ cells are correlated with different levels of platelet release. Blood.

[33] Liu Z.J., Hoffmeister K.M., Hu Z., Mager D.E., Ait-Oudhia S., Debrincat M.A., Pleines I., Josefsson E.C., Kile B.T., Italiano J. (2014). Expansion of the neonatal platelet mass is achieved via an extension of platelet lifespan. Blood.

[34] England J.M., Rowan R.M., Van Assendelft O.W., Bull B.S., Coulter W., Fujimoto K., Groner W., Richardson-Jones A., Klee G., Koepke J.A. (1993). Recommendations of the international council for standardization in haematology for ethylenediaminetetraacetic acid anticoagulation of blood for blood cell counting and sizing. Am. J. Clin. Pathol..

[35] Lombarts A.J.P.F., De Kieviet W. (1988). Recognition and prevention of pseudothrombocytopenia and concomitant pseudoleukocytosis. Am. J. Clin. Pathol..

[36] Vicari A., Banfi G., Bonini P.A. (1988). EDTA-Dependent pseudothrombocytopaenia: A 12-month epidemiological study. Scand. J. Clin. Lab. Investig..

[37] George J.N. (2000). Platelets. Lancelet.

[38] Prins M., van Leeuwen M.W., Teske E. (2009). Stability and reproducibility of ADVIA 120-measured red blood cell and platelet parameters in dogs, cats, and horses, and the use of reticulocyte haemoglobin content (CH(R)) in the diagnosis of iron deficiency. Tijdschr. Diergeneeskd..

